# Ascorbic acid concentrations in aqueous humor after systemic vitamin C supplementation in patients with cataract: pilot study

**DOI:** 10.1186/s12886-017-0515-2

**Published:** 2017-07-11

**Authors:** Young-Sool Hah, Hye Jin Chung, Sneha B. Sontakke, In-Young Chung, Sunmi Ju, Seong-Wook Seo, Ji-Myong Yoo, Seong-Jae Kim

**Affiliations:** 1 Biomedical Research Institute, Gyeongsang National University Hospital, Jinju, South Korea; 2 College of Pharmacy and Research institute of Pharmaceutical Sciences, Gyeongsang National University, Jinju, South Korea; 3 Department of Ophthalmology, Gyeongsang National University Hospital, Gyeongsang National University School of Medicine, Jinju, South Korea; 4 Gyeongsang Institute of Health Science, Gyeongsang National University, Jinju, South Korea; 5 Division of Pulmonology and Allergy, Department of Internal Medicine, Gyeongsang National University Hospital, Jinju, South Korea

**Keywords:** Antioxidant, Ascorbic acid, Aqueous humor, Cataract, Vitamin C

## Abstract

**Background:**

To measure ascorbic acid concentration in aqueous humor of patients with cataract after oral or intravenous vitamin C supplementation.

**Methods:**

Forty-two eyes of 42 patients with senile cataract who underwent uncomplicated cataract surgery were enrolled. Patients (*n* = 14 each) were administered oral vitamin C (2 g), intravenous vitamin C (20 g) or no treatment (control group) on the day before surgery. Samples of aqueous humor (0.1 cm^3^) were obtained by anterior chamber aspiration at the beginning of surgery and stored at −80 °C. Ascorbic acid concentration in aqueous humor was measured by high-pressure liquid chromatography.

**Results:**

The mean age at surgery was 62.5 years, with no difference among the three groups. The mean ± standard deviation concentrations of ascorbic acid in aqueous humor in the control and oral and intravenous vitamin C groups were 1347 ± 331 μmol/L, 1859 ± 408 μmol/L and 2387 ± 445 μmol/L, respectively. Ascorbic acid concentration was significantly lower in the control than in the oral (*P* < 0.01) and intravenous (*P* < 0.001) vitamin C groups and was significantly higher in the intravenous than in the oral vitamin C group (*P* < 0.05).

**Conclusions:**

Ascorbic acid concentration in aqueous humor is increased by systemic vitamin C supplementation, with intravenous administration being more effective than oral administration.

## Background

Vitamins are essential nutrients required for various biological processes in the body. Because they cannot be synthesized in the body, vitamins must be ingested in foods. Vitamin C (ascorbic acid) facilitates the conversion of cholesterol into bile acids and increases the absorption of iron in the gut. Vitamin C is also an antioxidant, protecting the body from the deleterious effects of free radicals, pollutants and toxins [1]. Deficiencies in vitamin C have been associated with anaemia, infections, bleeding tendency and delayed wound healing [2].

The concentration of ascorbate is about 15 times greater in the aqueous humor of the eye than in plasma, suggesting that vitamin C may protect against harmful factors within the eye [3]. However, the concentration of vitamin C in aqueous humor of patients with age-related cataract decreases with age of the patient (from 50 to 70 years old), suggesting that this decrease may play a role in susceptibility to cataract formation in older people [4, 5]. Vitamin C concentrations in aqueous humor are also lower in patients with various ophthalmic diseases. For example, the concentration of vitamin C in the anterior chamber has been reported lower in patients with Lowe’s syndrome and exfoliation syndrome than in age-matched controls [6–8]. Moreover, reduced levels of vitamin C in aqueous humor may be associated with glaucoma [8, 9]. Thus, measuring vitamin C concentrations in aqueous humor may be useful for studying the pathogenesis of several ocular diseases.

Systemic oral administration of 2.0 g of vitamin C resulted in saturation of aqueous humor, with additional vitamin C, up to 5 g, not further increasing its concentration [10]. That study, however, did not assess whether intravenously administered vitamin C resulted in higher concentrations in the anterior chamber. This study therefore investigated ascorbic acid concentration in aqueous humor, as measured by high-pressure liquid chromatography (HPLC), after systemic (oral and intravenous) vitamin C supplementation in patients with cataract.

## Methods

This study was approved by the Institutional Review Board of the Gyeongsang National University Hospital (no. 2016–04–012-002) and complied with the guidelines of the Declaration of Helsinki. All subjects provided written informed consent.

### Patients and sampling of aqueous humour

This study prospectively enrolled cataract patients with no previous ocular morbidities who had not undergone previous intraocular surgery or procedures. Patients with chronic systemic diseases (e.g., of the liver or kidneys), uncontrolled diabetes mellitus or hypertension, history of renal calculi or gout, hypersensitivity to vitamin C or history of vitamin C supplements were excluded, as were pregnant or lactating women. Participants were classified into control, oral vitamin C and intravenous vitamin C groups. The latter two groups were administered oral vitamin C (2 g/day) or intravenous vitamin C (20 g/day) on the day before cataract surgery. To minimize the effect of time on ascorbic acid concentration, 1 g (oral group) or 10 g (intravenous group) of vitamin C was administered twice at 8 h interval to complete the total dose of 2 g or 20 g to the patients on the day before surgery. And, on the day of surgery, the collection of aqueous humor was completed within at least 2 h from 8 am. Samples of aqueous humor (0.1 cm^3^) were obtained by anterior chamber aspiration into a syringe using a 26-gaugeze needle at the beginning of the surgery, prior to the injection of viscoelastic. All samples were stored at −80 °C in amber tubes.

### Materials

L-Ascorbic acid and metaphosphoric acid (MPA) were purchased from Sigma-Aldrich Co. (St Louis, MO, USA) and Kanto Chemicals Co. Inc. (Tokyo, Japan), respectively. HPLC-grade acetonitrile and water were purchased from Fisher Scientific (Pittsburgh, PA, USA). All other chemicals were of analytical grade.

### Measurement of ascorbic acid in aqueous humour

**Fig. 1:**
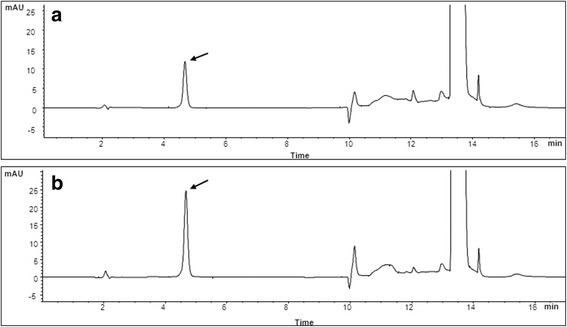
Representative HPLC chromatograms after stabilization of vitamin C. **a** vitamin C standard solution (1000μM) (**b**) human aqueous sample collected after intravenous administration of vitamin C. The arrows indicate vitamin C. UV absorption at 265nm is shown in milliabsorption units (mAU)

Ascorbic acid concentrations in aqueous humour were measured by HPLC, as previously described with slight modifications [11]. Briefly, 100 μL of cold 10% MPA solution was added to a 100 μL aliquot of sample, vortexed and kept at 4 °C for 10 min for protein precipitation and ascorbic acid stabilization. The samples were centrifuged at 4 °C for 5 min at 10,000 g, and 20 μL of supernatant was transferred to a clean tube and diluted with 180 μL of 0.9% MPA. A 10 μL aliquot of each sample was injected into the HPLC. All solutions were carefully protected from light during sample preparation and analysis. Ascorbic acid was determined using an Agilent 1260 HPLC system (Agilent, Singapore) and a Synergi Hydro-RP column (4 μm, 4.6 × 150 mm; Phenomenex, CA, USA) maintained at 20 °C. The mobile phase consisted of 0.9% MPA (A) and acetonitrile (B), with gradient elution at a flow rate of 0.7 mL/min. The initial composition of 100% A was kept for 5 min, increased from 0% to 90% B for 3 min and maintained at 90% B for 2 min. The gradient was then changed back to the initial condition over 1 min and kept at the initial condition for 6 min. The total run time was 17 min. Effluent was monitored using a UV detector set at 265 nm. Representative HPLC chromatograms after stabilization of vitamin C are presented in Fig. 1. The calibration curves of ascorbic acid were linear over the ranges studied, with *r* ^2^ > 0.995.

Data were summarized using mean and standard deviation. Statistical analyses comparing three groups were performed using a one-way analysis of variance (ANOVA), and post hoc analysis with Bonferonni correction was used to evaluate the difference between the two groups using SPSS ver 18.0 (SPSS Inc., Chicago, IL, USA). Statistical significance was set at 0.05.

## Results

Table 1 shows the baseline characteristics of the 42 study subjects (42 eyes) enrolled from February to July 2015. The mean age of this cohort was 62.5 ± 10.1 years. Mean age, gender and status of cataract (Lens Opacities Classification System III, LOCS III) were similar (*P* > 0.05) in the three groups.

**Table 1:** Clinical characteristics of the study population

		Intravenous vitamin C	Oral vitamin C	Control	*P* value	Total
Number of patients		14	14	14		42
Age, yr (mean±SD)		64 ± 10.9	62.6 ± 10.3	60.5 ± 6.7	0.541	62.5 ± 10.1
Sex (M/F)		7/7	6/8	8/6	0.741	21/21
Grade of Cararact (LOCS III)	NO2NC2	0	1	1		2
	NO3NC3	8	8	9		25
	NO4NC4	5	4	4		13
	NO5NC5	1	1	0		2

*LOCS* Lens Opacities Classification System, *NO* Nuclear Opalescence, *NC* Nuclear Color

**Fig. 2:**
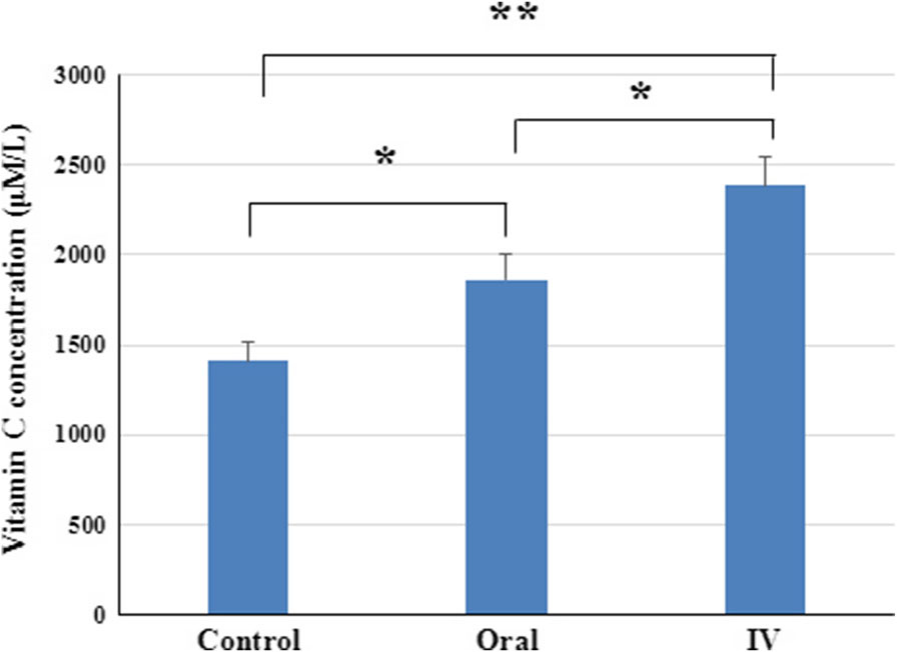
Ascorbic acid concentrations in the aqueous humor of the control, oral vitamin C and intravenous vitamin C groups. *P < 0.05, **P < 0.01

Ascorbic acid concentrations in aqueous humour are presented in Fig. 2. The mean ± standard deviation concentrations of ascorbic acid in the aqueous humor of the control, oral vitamin C and intravenous vitamin C groups were 1347 ± 331 μmol/L, 1859 ± 408 μmol/L and 2387 ± 445 μmol/L, respectively. Ascorbic acid concentration was significantly lower in the control than in the oral (*P* < 0.01) and intravenous (*P* < 0.001) vitamin C groups and was significantly higher in the intravenous than in the oral vitamin C group (*P* < 0.05).

## Discussion

To our knowledge, no previous study has investigated the effects of oral and intravenous vitamin C supplementation on ascorbic acid concentrations in aqueous humor. Our results indicate that both types of systemic vitamin C supplementation increased ascorbic acid concentrations in aqueous humor, with intravenous administration being more effective than oral administration.

Previous studies show that ascorbic acid concentrations are much higher in aqueous humor than in plasma [12, 13]. This concentration gradient is a result of active transport in the ciliary epithelium [14]. In eyes, ascorbic acid protects against the effects of ultraviolet rays and oxidants, thereby preventing cataract formation [5, 15]. Lower than normal ascorbic acid concentrations in aqueous humor have been reported in various ophthalmic diseases. For example, the concentration of vitamin C was reported to decrease with age in patients with age-related cataract, suggesting that reductions in ascorbic acid may play an important role in cataract formation [4, 5]. Lower levels of vitamin C in aqueous humor may be associated with glaucoma, including in patients with primary open angle glaucoma and secondary glaucoma [8, 9, 16]. Finally, patients with Lowe’s syndrome and exfoliation syndrome have significantly lower levels of ascorbic acid in aqueous humor than age-matched controls [6, 8]. These findings suggest that vitamin C concentrations may provide clues to the pathogenesis and treatment of several ocular diseases.

High ascorbic acid concentrations in aqueous humor may protect the lens against the cataractogenic effects of UV radiation [17, 18]. Moreover, oral, topical or intravenous application of vitamin C lowers intraocular pressure in glaucoma patients [8, 9, 19]. The Age-Related Eye Disease Study (AREDS) found that long-term supplementation with vitamin C (500 mg/day) and other vitamins was effective in retarding the progression of age-related macular degeneration (AMD) [20] and in delaying the progression of lens opacities [4, 18]. Supplementation with low- and high-dose vitamin C was found to be associated with decreased risk of glaucoma [21]. However, the optimal concentrations and routes of administration remain unclear.

Our findings are largely consistent with those of earlier studies. A 10% increase in plasma ascorbate concentration was found to increase ascorbate concentrations in aqueous humor by 48% [13] and 66% [22]. Oral administration of 2.0 g of vitamin C was found to saturate the aqueous humor, with further vitamin C supplementation having no effect on its concentration in aqueous humor [10]. By contrast, this study showed that intravenous administration was more effective than oral administration at increasing the ascorbic acid concentration in aqueous humor. Intravenous supplementation with high-dose vitamin C and its increased concentration in aqueous humor may protect normal ocular structures against harmful reactive oxygen radicals and may treat diseases associated with these radicals. Our previous study found that systemic (oral or intravenous) vitamin C supplementation reduced the size of corneal opacities resulting from infectious keratitis, with intravenous vitamin C being more effective than oral vitamin C [23]. Additional studies are needed to determine the effect of vitamin C in patients with severe uveitis, glaucoma, cataract and other inflammatory diseases.

This study had several limitations, including its small sample size. In addition, serum concentrations of vitamin C were not measured. And, the subjects were not randomly divided into three groups, so there could be a selections bias. Finally, there is no standardization of ascorbic intake of the individual patients, so, it can affect ascorbic acid level in the aqueous humor. Future studies assessing the impact of vitamin C on various ocular diseases should include direct administration of various concentrations of vitamin C and measurements of vitamin C in aqueous humor. Large, long-term clinical studies are warranted to establish the optimal dose, route of administration, duration of treatment and frequency of administration of vitamin C for various ophthalmic diseases.

## Conclusion

In conclusion, the results of this study suggest that systemic administration of vitamin C increased ascorbic acid concentration in aqueous humor, with intravenous administration being more effective than oral administration.

## Abbreviations

AREDS: Age-Related Eye Disease Study
HPLC: High-pressure liquid chromatography
LOCS: Lens opacities classification system

## References

[1] Buettner GR, Schafer FQ (2006). Albert Szent-Gyorgyi. Vitamin C identification. Biochemist.

[2] Graumlich JF, Ludden TM, Conry-Cantilena CLR, Wang Y, Levine M (1997). Pharmacokinetic model of ascorbic acid in healthy male volunteers during depletion and repletion. Pharm Res.

[3] Reiss GR, Werness PG, Zollman PE, Brubaker RF (1986). Ascorbic acid levels in the aqueous humor of nocturnal and diurnal mammals. Arch Ophthalmol.

[4] Wei L, Liang G, Cai C, Ly J (2016). Association of vitamin C with the risk of age-related cataract: a meta-analysis. Acta Ophthalmol.

[5] Canadananovic V, Latinovic S, Barisic S, Babic N, Jovanovic S (2015). Age-related changes of vitamin C level in aqueous humor. Vojnosanit Pregl.

[6] Hayasaka S, Yamada T, Nitta K, Kaji Y, Hiraki S, Tachinami K, Matsumoto M, Yamammoto S, Yamammoto S (1997). Ascorbic acid and amino acid values in the aqueous humor of a patient with Lowe’s syndrome. Graefes Arch Clin Exp Ophthalmol.

[7] Ferreira SM, Lerner SF, Brunzini R, Evelson PA, Llesuy SF (2009). Antioxidant status in the aqueous humor of patients with glaucoma associated with exfoliation syndrome. Eye (Lond).

[8] Koliakos GG, Kontas AG, Schlotzer-Scherhardt U, Bufidis T, Georgiadis N, Ringvold A (2002). Ascorbic acid concentration is reduced in the aqueous humor of patients with exfoliation syndrome. Am J Ophthalmol.

[9] Leite MT, Prata TS, Kera CZ, Miranda DV, de Moraes Barros SB, Melo LA (2009). Ascorbic acid concentration is reduced in the secondary aqueous humor of glaucomatous patients. Clin Experiment Ophthalmol.

[10] Iqbal Z, Midgley JM, Watson DG, Karditsas SD, Dutton GN, Wilson WS (1999). Effect of oral administration of vitamin C on human aqueous humor ascorbate concentration. Zhongguo Yao Li Xue Bao.

[11] Ross MA (1994). Determination of ascorbic acid and uric acid in plasma by high-performance liquid chromatography. J Chromatogr B Biomed Appl.

[12] Badhu B, Baral N, Lamsal M, Das H, Dhital BA (2007). Plasma and aqueous humor ascorbic acid levels in people with cataract from diverse geographical regions of Nepal. Southeast Asian J Trop Med Public Health.

[13] Taylor A, Jacques PF, Nowell T, Perrone G, Blumberg J, Handelman G, Jozwiak B, Nadler D (1997). Vitamin C in human and guinea pig aqueous, lens and plasma in relation to intake. Curr Eye Res.

[14] Kong CW, Chan CY, Shahidullah M, Do CW, To CH (2002). The mechanism of aqueous humor formation. Clin Exp Optom.

[15] Babizhayev MA (2011). Mitochondria induce oxidative stress, generation of reactive oxygen species and redox state unbalance of the eye lens leading to human cataract formation: disruption of redox lens organization by phospholipid hydroperoxides as a common basis for cataract disease. Cell Biochem Funct.

[16] Xu P, Lin Y, Porter K, Liton PB (2014). Ascorbic acid modulation of iron homeostasis and lysosomal function in trabecular meshwork cells. J Ocul Pharmacol Ther.

[17] Reddy VN, Giblin FJ, Lin LR, Chakrapani B (1998). The effect of aqueous humor ascorbate on ultraviolet-B-induced DNA damage in lens epithelium. Invest Ophthalmol Vis Sci.

[18] Age-Related Eye Disease Study Research Group (2001). A randomized, placebo-controlled, clinical trial of high-dose supplementation with vitamins C and E and beta carotene for age-related cataract and vision loss: AREDS report no. 9. Arch Ophthalmol.

[19] Lee PF, Fox R, Henrick I, Lam WK (1978). Correlation of aqueous humor ascorbate with intraocular pressure and outflow facility in hereditary buphthalmic rabbits. Invest Ophthalmol Vis Sci.

[20] Chew EY, Clemons TE, Agron E, Sperduto RD, Sangiovanni JP, Davis MD, Ferris FL (2014). 3rd; age-related eye disease study research group. Ten-year follow-up of age-related macular degeneration in the age-related eye disease study AREDS report no.36. JAMA Ophthalmol.

[21] Wang SY, Singh K, Lin SC (2013). Glaucoma and vitamins a, C, and E supplement intake and serum levels in a population-based sample of the United States. Eye (Lond).

[22] Taylor A, Jacques PF, Nadler D, Morrow F, Sulsky SI, Shepard D (1991). Relationship in humans between ascorbic acid consumption and levels of total and reduced ascorbic acid in lens, aqueous humor, and plasma. Curr Eye Res.

[23] Cho YW, Yoo WS, Kim SJ, Chung IY, Seo SW, Yoo JM (2014). Efficacy of systemic vitamin C supplementation in reducing corneal opacity resulting from infectious keratitis. Medicine.

